# Study on the mechanism of acute liver injury protection in Rhubarb anthraquinone by metabolomics based on UPLC-Q-TOF-MS

**DOI:** 10.3389/fphar.2023.1141147

**Published:** 2023-03-06

**Authors:** Xiaohong Gong, Fang Zhang, Yunxia Li, Cheng Peng

**Affiliations:** Chengdu University of Traditional Chinese Medicine, Chengdu, China

**Keywords:** Rhubarb anthraquinone, acute liver injury, metabolomics, UPLC-Q-ToF-MS, traditional Chinese medicine

## Abstract

As a traditional Chinese medicine, rhubarb has been used in a variety of liver diseases and it is widely used in clinic to prevent and treat acute liver injury. Anthraquinone, as the main medicinal component of rhubarb, can reverse the further development of liver fibrosis caused by acute liver injury. In this study, metabonomics was used to explore the mechanism of different doses of rhubarb anthraquinone on acute liver injury in rats. Rhubarb anthraquinone was administered intragastric to rats at doses of 3.9, 7.8 and 15.6 mg/kg, respectively, for 7 days, and then 30% CCl_4_ was injected intraperitoneally at the dose of 1 ml/kg to replicate the acute liver injury model. The biochemical indicators content of ALT, AST, ALP, γ-GT, TG, TC, LDL, HDL in serum and GSH, Hyp, SOD, TNF-α, IL-6 and IL-8 in liver tissue extract were tested respectively, and liver tissue was histopathologically analysis. At the same time, UPLC-Q-TOF-MS combined with non-targeted metabolomics were used to study the metabolites and metabolic pathways of rhubarb anthraquinone in treating acute liver injury. Compared with normal rats, the contents of ALT, AST, ALP, TG, TC, LDL, γ-GT in serum and Hyp, MDA, IL-6, IL-8, TNF-α in the liver tissue extract were significantly increased in model rats (*p* < 0.05, *p* < 0.01), and the content of HDL in the serum was significantly decreased (*p* < 0.05); the activities of GSH and SOD in liver tissue extract were also significantly decreased (*p* < 0.05). After administration of rhubarb anthraquinone, compared with the model group, with the increase of dosage, some biochemical indexes showed opposite changes, and gradually approached to normal rats. 12 different metabolites were identified by metabonomics, and the biosynthesis and metabolism of phenylalanine, tyrosine and tryptophan, the metabolism of amino sugars, nucleotide sugars and pyrimidines metabolism, and the biosynthesis of steroid hormone were identified based on the biomarker analysis. Based on the biochemical analysis and metabonomics analysis of rats with acute liver injury treated with different doses of rhubarb anthraquinone, combined with histopathological observation, the results show that the protective effect of rhubarb anthraquinone on acute liver injury is related to the dosage; Meanwhile, the metabolic pathway analysis suggested that rhubarb anthraquinone alleviate acute liver injury by regulating inflammation, oxidative stress and fibrosis disorders. This study explained the therapeutic effect of rhubarb anthraquinone on acute liver injury from both material basis and action pathway, and provided safe and effective research ideas for clinical application of rhubarb.

## Introduction

Acute liver injury (ALI) is a series of liver diseases caused by alcoholism, drug-induced, autoimmune diseases, viral infections, and radiation damage (Riordan and Wiliams, 2003; Wu et al., 2010; Frank et al., 2020). As a common clinical critical emergency, ALI has a poor prognosis and can further induce liver fibrosis and even liver cancer. The pathogenesis and pathological mechanism of ALI are relatively complex, with different initiating factors and persistent pathogenic factors in multiple stages, which involve the correlation and influence between multiple organs and multiple systems. Therefore, it is often difficult to achieve good results with a single treatment method (Thawley., 2017; Crismale and Friedman, 2020; Liu et al., 2021). So far, there are no definite curative, safe and stable therapeutic drugs for treatment of AIL in the world. It is of great significance to explore effective treatments for acute liver injury, and in-depth study of its pathogenesis is crucial for formulating effective AIL treatment strategies.

As a treasure trove of medical resources, traditional Chinese medicine has great potential for development, and its multi-component, multi-target and multi-approach functions provide more possibilities for the treatment of acute liver injury. According to the “Guidelines for the Diagnosis and Treatment of Liver Fibrosis with Integrated Traditional Chinese and Western Medicine” formulated by the Liver Disease Professional Committee of the Chinese Association of Integrative Medicine, rhubarb is the preferred Chinese medicine for the prevention and treatment of the disease (Lv and Ma, 2016; Li et al., 2019; Professional Committee of Liver Diseases of the Chinese Society of Integrative Medicin., 2019). Combined with the treatment strategy of acute liver injury, studies have shown that rhubarb downregulated the expression of tumor necrosis TNF-α and transforming growth TGF-β1, upregulated the expression of IFN-γ, and inhibited the activation and proliferation of HSC, thus further reduced the synthesis and expression of α-SMA, and inhibited the activation and transformation of lipocyte into myofibroid cells (Xue et al., 2015; Zhang et al., 2016; Zhang et al., 2017). At the same time, by increasing the expression of TIMP-1, decreasing the expression of MMP-1, MMP-2 and CTGF, and reducing the content of proline hydroxylase and MAO. So as to reduce lipid peroxidation, promote collagen decomposition and reduce collagen production (Chen et al., 2010; Wei et al., 2016). Thus, rhubarb anthraquinone, which is a group of medicinal components, has the pharmacological effect of reversing the further development of acute liver injury (Arosio et al., 2000; Neyrinck et al., 2017).

Metabolomics is a discipline that studies the changes of endogenous metabolites in the body. It mainly analyzes the changes of metabolite expression profiles before and after drug action by using liquid chromatography-mass spectrometry, gas chromatography-mass spectrometry and nuclear magnetic resonance. At the same time, combining pattern recognition and other chemoinformatics techniques to analyze differences in metabolic fingerprints, to screen biomarkers with differential changes, and to further speculate on the process of biochemical changes in the body (Li et al., 2014; Su et al., 2014). Different from traditional efficacy/toxicity research methods, metabolomics can discover drug efficacy/toxicity substances and their effect rules faster and more accurately, and has a good expression for the comprehensive pharmacological effects of traditional Chinese medicines with multiple targets and multiple pathways (Zhang et al., 2016).

In order to clarify the mechanism of rhubarb anthraquinone in hepatoprotective effection, UPLC-Q-TOF-MS combined with non-targeted metabolomics was used in this study to compare the differences in serum metabolic profiles of rats with acute liver injury before and after administration of rhubarb anthraquinone, and the characteristic biomarkers that cause metabolic disorders in serum samples were screened. The metabolic pathway analysis of rhubarb anthraquinone in the treatment of acute liver injury was conducted by biomarkers combined with biochemical indexes, and the comprehensive evaluation model of rhubarb anthraquinone concentration-effect was established, so as to provide reference for further guiding the rational drug use of rhubarb in clinical practice.

## Experimental contents

### Chemicals and reagents

Rhubarb anthraquinone (the anthraquinone plant extraction rate 1.2%, and the content is 80% calculated from rhein, emodin, chrysophanol, physcion and aloe-emodin), was purchased from Chengdu Croma Biological Co., Ltd (Batch Number: CHB2021546). Methanol, acetonitrile, formic acid and water (HPLC grade) were purchased from Fisher Co. (Pittsburg, PA, United States). Other chemicals were all analytical grade, purchased from Cologne Chemical Reagent Factory (Chengdu, China).

### Animal handling and animal experiment design

A total of 50 SPF level Sprague-Dawley rats, half male and half female, weighing approximately 200–240 g were purchased from Chengdu Dashuo Animal Co., Ltd (Certification Number: SCXK(川)2020–030) and standby after passing quarantine. Before the formal experiment, the rats were raised in the laboratory animal room of the Pharmacological Experiment Center for 1 week in advance to adapt to the environment, with a 12 h day-night cycle was kept, the room temperature was regulated at (24 ± 2)°C and the humidity was at 60–70% and the animals were provided free access to water and standard diet.

Rats were randomly divided into five groups, including normal control group (NC), acute liver injury group (ALI), rhubarb anthraquinone low-dose group (RA-L), rhubarb anthraquinone medium-dose group (RA-M) and rhubarb anthraquinone high-dose group (RA-H), and 10 rats in each group. Among them, the rats in NC and ALI were given 20 ml/kg/day of normal saline by intragastric administration, and the RA-L, RA-M, RA-H were given the same dose of rhubarb anthraquinone at the concentrations of 3.9 mg/kg, 7.8 mg/kg, and 15.6 mg/kg (the anthraquinone was dissolved by 0.5% CCMC-Na, then diluted with normal saline into 1.3 mg/ml, 2.6 mg/ml, 3.9 mg/ml, and administration at the dosage of 3 ml/kg), for seven consecutive days. One hour after the last administration, rats in the ALI and each administration group were intraperitoneally injected with 30% CCl_4_ olive oil solution at a dose of 1 ml/kg to establish an acute liver injury model, while the rats in NC were only injected with the same dose of olive oil. All rats were treated in accordance with the Guidelines for the Management and Use of Experimental Animals of Chengdu University of Traditional Chinese Medicine (Approval Number: 2021–67).

### Collection of plasma samples

After the model was established, the rats were fasted but drank freely for 24 h 30 min after the last dose, rats were anesthetized with 10% chloral hydrate, blood was collected from abdominal aorta, and the serum samples were obtained by separating the supernatant from the blood centrifugation with 3,500 rpm for 10 min after 30 min of coagulation at 4°C and stored in the refrigerator at −80°C immediately. Large part of the serum was used for blood biochemical analysis, and other were used for metabolomics analysis. The livers were removed from the rats immediately after sacrifice. Rats were dissected after cervical dislocation, and the liver tissue were removed and rinsed with cold normal saline. Some of them were quantitatively homogenized for biochemical analysis, and others for pathological analysis.

### Serum biochemistry and histopathological analysis

According to the contents of the kit, the levels of aspartate aminotransferase (AST), alanine aminotransferase (ALT), alkaline phosphatase (ALP), Glutamyl transpeptidase (γ-GT), triglycerides (TG), total cholesterol (TC), low-density lipoprotein (LDL) and high-density lipoprotein (HDL) levels in serum, and Glutathione (GSH), hydroxyproline (Hyp), superoxide dismutase (SOD), malondialdehyde (MDA), tumor necrosis factor-alpha (TNF-ɑ), interleukin 6 (IL-6) and interleukin 8 (IL-8) in liver tissues were measured with an Mindray BS-360s automatic biochemistry Analyzer (Shanghai Fuzhong Biosciences Corporation, China). Liver tissue were preserved in 10% neutral formaldehyde for dehydration fixation, processing and trimming, and then embedded in paraffin, sectioned to a thickness of approximately 5μm, and stained with hematoxylin and eosin.

### Pretreatment of plasma samples

After thawed, the serum samples were subjected to protein precipitation with methanol. 300 μl serum samples were transferred into a 1.5 ml polypropylene tube with 900 µl methanol, and then mixing for 2 min. The supernatant samples were collected after centrifuged at 12,000 rpm for 10 min at 4°C and transferred to a new 1.5 ml polypropylene tube and filtered through 0.22 µm filter. At the same time, two samples with equal volume were randomly selected in each group as the quality control sample (QC), which contained most of the information about all the samples. During the whole injection process, one QC injection was performed for every 10 samples, which can be used as UPLC-Q-TOF-MS optimized conditions.

### Chromatography and mass spectrometry conditions

Thermo Ultimate 3000 UPLC-Q-TOF-MS was applied to this analysis. For liquid spectrometry, the analysis was conducted with a Hypersil GOLD C18 analytical column (2.1 mm*150 mm, 1.9 μm), and the mobile phases of solvent A (Water spiked with 0.1% formic acid) and solvent B (Acetonitrile) with gradient elution as follows: a linear gradient of 2% B over initial 1 min, 2–50% B over 1.0 min–12.0 min, 50–98% B over 12.0 min–15.0 min, 98–2% B over 15.0 min–18.0 min, 2% B over 18.0 min–20.0 min. The flow rate was set as 0.25 ml/min, the column temperature maintained at 40°C, and 5 µl supernatant samples were used for analysis.

Thermo Q Exactive quadrupole for mass spectrometry, and the electrospray ionization source in both positive and negative mode was used. The electrospray source parameters were set as follows: electrospray voltage was 3.5 kV in positive ionization mode and 2.5 kV in negative, capillary temperature was 325°C and the source temperature was 600°C, the nebulizer was 45 pisg for positive and 35 pisg for negative respectively. The full scan mode was used with a scan range of 80–1,500 Da, HCD for secondary fragmentation with a collision voltage of 30 eV and dynamic exclusion to remove unnecessary MS/MS information.

### Data processing and pattern recognition analysis

The methods of chemometrics and multivariate statistics were used for dimensionality reduction and classification analysis of the collected data, so as to mine and extract the most useful information. Data preprocessing is performed at frist, including converted the raw. wifi data into. mzXML through Proteowizard software; XCMS program (www.bioconductor.org/) performed processing steps such as peak identification, filtration, and alignment on the original data, and obtained matrix including m/z, retention time, and intensity data, and finally exported and saved.

The multivariate statistical analysis method was used to the further study the phenotype of the serum metabolome, so as to better observe and distinguish the subtle differences in the metabolic profiles of the NC, ALI and each administration group. The data matrices were imported into the SIMCA-P 14.0 (Umetrics, Umeå, Sweden) software for multivariate statistical analysis, including the unsupervised statistical method of principal component analysis (PCA) was used to perform the differential analysis of metabolic components in each group, and the supervised statistical analysis of principal least-squares discriminant analysis (PLS-DA) was used to show the differences between the NC, ALI, RA-L, RA-M and RA-H, a permutation test was used to prevent overfitting of the PLS-DA analytical model at the same time. The interpretability and predictability of the model were evaluated by cross-validated R_2_Y and Q_2_ variable values, and the Potential variables were selected by the variable projection importance (VIP) value >1.0 in the OPLS-DA model. SPSS 26.0 (Chicago, IL, United States) software was used to conduct *t*-test and One-way analysis of variance (ANOVA) analysis for these latent variables, and *p* < 0.05 was considered to be statistically significant to screen out the differential metabolites.

### Biomarker identification and metabolic pathway analysis

UPLC-Q-TOF-MS/MS was used to accurately determine the molecular ions of the screened differential compounds, the fragments information obtained in MS/MS mode were tentatively matched and identified through the online database such as Metlin (http://metlin.scripps.edu), HMDB (http://www.hmdb.ca) and Massbank (and to obtain the accurate metabolite informations. According to set 20 ppm as the accepted mass error to further confirm the biomarkers, the accurate metabolite informations was obtained. The pathway library of *Rattus norvegicus* (rat) in MetaboAnalyst 5.0 (http://www.metaboanalyst.ca/) was used to analyze the metabolic pathways of different doses of rhubarb anthraquinone for hepatoprotective effects. Through enrichment and topology analysis, the possible metabolic pathways of bioturbation were identified, and then, the analysis of differential metabolites between groups was performed to draw a metabolic pathway association network diagram.

## Results

### Behavioral observations

The hepatoprotective effects of different doses of rhubarb anthraquinone on acute liver injury were analyzed by the calculation of the formula (organ coefficient/% = organ weight/rat body weight*100%), and the results are shown in Table 1. Compared with the NC, the rat liver coefficient in ALI were significantly increased (*p* < 0.01); Compared with the ALI, the rats liver coefficient in RA-H were significantly decreased (*p* < 0.05), while the rats liver coefficient of RA-M and RA-L decreased to varying degrees, but there were no significant difference (*p* > 0.05).

**TABLE 1:** The organ weight and organ coefficient of liver in each group (Mean ± *s*).

Group	W/g	Liver	
		W/g	Coe %
NC	243.53 ± 2.43	8.65 ± 1.20	3.56 ± 0.25
ALI	253.82 ± 2.61	13.28 ± 2.47*	5.23 ± 0.89**
AR-L	247.42 ± 2.14	13.62 ± 2.18	5.11 ± 0.64
AR-M	241.37 ± 2.78	11.61 ± 1.59	4.82 ± 0.38^#^
AR-H	235.65 ± 1.83	10.83 ± 1.75	4.61 ± 0.54^#^

Compared with the NC, *p < 0.05, **p < 0.01; compared with the ALI.

#*p* < 0.05.

### Biochemical analysis and histopathological observations

**FIGURE 1:**
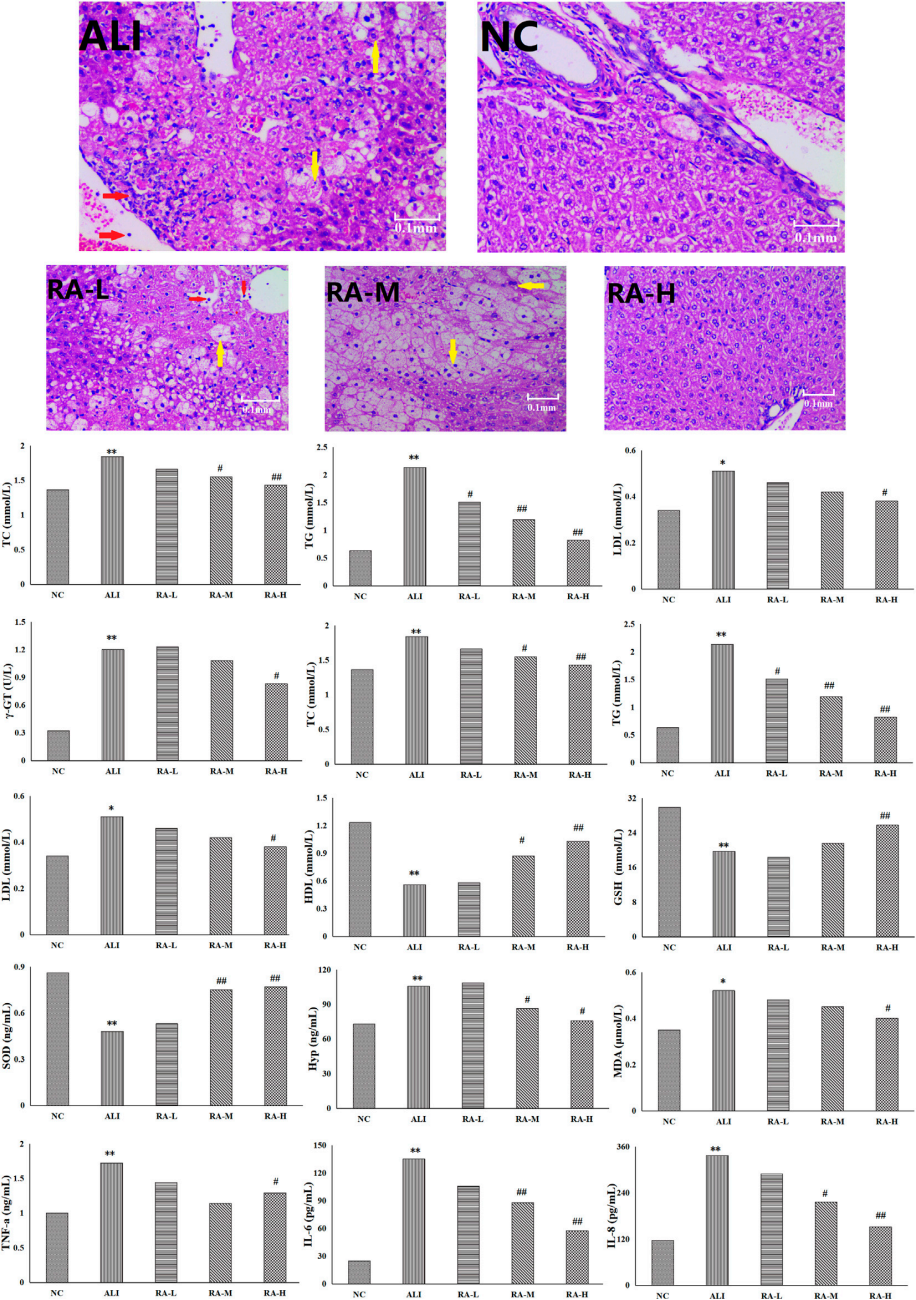
The effects of rhubarb anthraquinone on liver tissue of rats with acute liver injury refers to balloon degeneration and punctate necrosis of hepatocytes; refers to the infiltration of lymphocytes and neutrophils.

Compared with the NC, the rat serum contents of ALT, AST, ALP, TG, TC, LDL and γ-GT in ALI were significantly increased (*p* < 0.05, *p* < 0.01), and the HDL content was significantly decreased (*p* < 0.05). After administration of rhubarb anthraquinone, the rat contents of ALT, AST, ALP, TG, TC, LDL and γ-GT in RA-H were significantly decreased compared with ALI (*p* < 0.05, *p* < 0.01), and HDL was significantly increased (*p* < 0.01). Some indexes in RA-L and RA-M also showed significant changes, and the results were shown in Table 2 and Figure 1.and Figure 1.

**TABLE 2:** The effects of rhubarb anthraquinone on serum biochemical indices in rats with acute liver injury (Mean ± *s*).

Index	NC	ALI	RA-L	RA-M	RA-H
ALT (U/L)	11.84 ± 4.0	54.0 ± 36.13**	48.08 ± 19.34	43.18 ± 28.96^#^	23.17 ± 1.57^##^
AST (U/L)	95.34 ± 14.81	253.75 ± 88.52**	274.1 ± 87.25	190.66 ± 10.91^##^	120.06 ± 43.54^##^
ALP(U/L)	136.32 ± 28.64	333.32 ± 19.38^**^	307.26 ± 74.80	299.4 ± 54.35	162.48 ± 52.17^##^
γ-GT (U/L)	0.32 ± 0.09	1.2 ± 0.31^**^	1.23 ± 0.18	1.08 ± 0.28	0.83 ± 0.08^#^
TC (mmol/L)	1.36 ± 0.09	1.84 ± 0.12**	1.66 ± 0.14	1.55 ± 0.16^#^	1.43 ± 0.14^#^
TG (mmol/L)	0.63 ± 0.11	2.13 ± 0.50**	1.51 ± 0.31^#^	1.19 ± 0.21^##^	0.82 ± 0.23^##^
LDL (mmol/L)	0.34 ± 0.02	0.51 ± 0.05*	0.46 ± 0.04	0.42 ± 0.03	0.38 ± 0.06^#^
HDL (mmol/L)	1.23 ± 0.08	0.56 ± 0.12**	0.58 ± 0.05	0.87 ± 0.11^#^	1.03 ± 0.12^##^

Compared with the NC, *p < 0.05, **p < 0.01, compared with the ALI.

#*p* < 0.05.

##*p <* 0.01.

Compared with the NC, the rat liver tissue activities of Hyp, MDA, IL-6, IL-8 and TNF-α in ALI were increased significantly (*p* < 0.05, *p* < 0.01), while the activities of GSH and SOD were decreased significantly (*p* < 0.05, *p* < 0.01). After administration of rhubarb anthraquinone, the rat liver tissue activities of Hyp, MDA, TNF-α and IL-6 in RA-H were significantly decreased (*p* < 0.05, *p* < 0.01), while the GSH and SOD activities were significantly increased (*p* < 0.05, *p* < 0.01). Some biochemical indexes of liver function in RA-L and RA-M also showed obvious changes, and the results were shown in Table 3

**TABLE 3:** The effects of rhubarb anthraquinone on liver biochemical indices in rats with acute liver injury (Mean ± *s*).

Index	NC	ALI	RA-L	RA-M	RA-H
GSH (mmol/L)	29.86 ± 3.14	19.71 ± 1.73^**^	18.33 ± 1.25	21.57 ± 1.73	25.73 ± 3.79^##^
SOD (ng/mL)	0.86 ± 0.04	0.48 ± 0.03^**^	0.53 ± 0.08	0.75 ± 0.08^##^	0.77 ± 0.12^##^
Hyp (ng/mL)	72.87 ± 3.40	105.51 ± 7.73^**^	108.53 ± 5.62	86.31 ± 4.47^#^	75.75 ± 8.59^#^
MDA (μmol/L)	0.35 ± 0.01	0.52 ± 0.03^*^	0.48 ± 0.03	0.45 ± 0.02	0.40 ± 0.03^#^
TNF-a (ng/mL)	1.00 ± 0.11	1.72 ± 0.34^**^	1.44 ± 0.22	1.14 ± 0.13	1.29 ± 0.16^#^
IL-6 (pg/mL)	24.81 ± 6.41	135.02 ± 13.62^**^	105.59 ± 15.61	87.89 ± 11.33^##^	57.34 ± 6.54^##^
IL-8 (pg/mL)	116.69 ± 12.64	337.02 ± 12.43^**^	289.23 ± 46.86	216.34 ± 45.81^#^	151.60 ± 27.72^##^

Compared with the NC, *p < 0.05, **p < 0.01, compared with the ALI, ^#^ *p* < 0.05, ^##^ *p <* 0.01.

### Histopathological observations

After the liver tissue was stained with hematoxylin-eosin, the changes of hepatocytes in each group was observed by electron microscope magnification 400 times. The hepatocytes in the NC were normal in shape, neatly arranged, and had no vacuolar lesions and lipid droplets; however, the whole arrangement of hepatocytes in the ALI were disordered, the volume increased, and some hepatocytes showed balloon-like degeneration and spot-like necrosis. The lymphocyte and neutrophil infiltration, and even present obvious vacuoles. After the administration of rhubarb anthraquinone, compared with the ALI, the morphology of hepatocytes in each administration group were significantly improved with the increase of administration dose, which showed that the cells were arranged more neatly and the degree of steatosis was less. The results were shown in Figure 1.

### PCA and PLS-DA analysis and model verification

**FIGURE 2:**
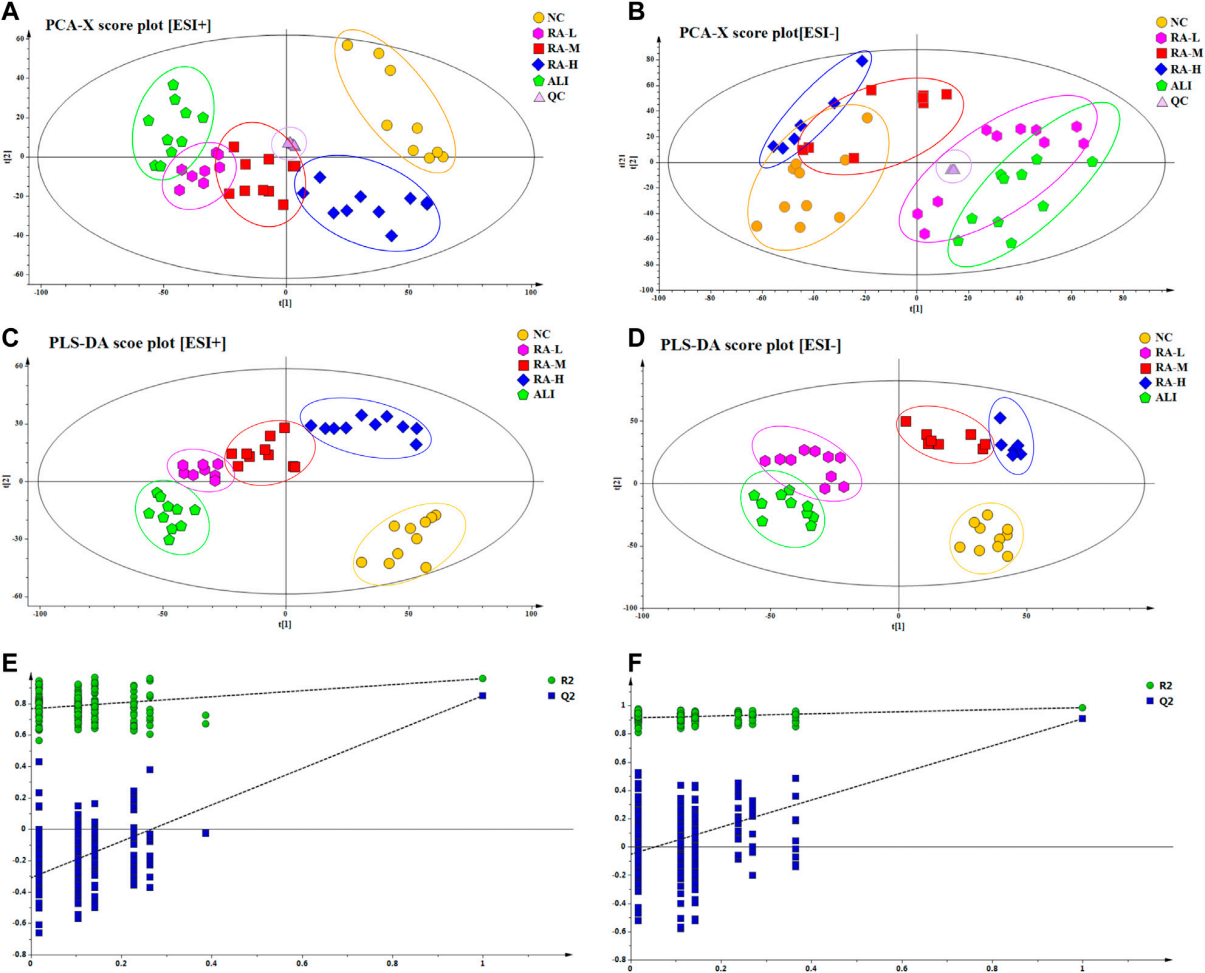
PCA score plots and PLS-DA score plots of different groups in ESI+ and ESI- mode respectively. The score plots of PCA of NC, ALI, RA-L, RA-M and RA-H groups from PCA analysis in ESI+ **(A)** and ESI- **(B)**; The score plots of PLS-DA of NC, ALI, RA-L, RA-M and RA-H groups from PLS-DA analysis in ESI+ **(C)** and ESI- **(D)**; 200-permutation test of PLS-DA model in ESI+ **(E)** and ESI- **(F)**.

A variety of pattern recognition methods such as PCA and PLS-DA were used to phenotype the metabolic profiles of rhubarb anthraquinone before and after administration, so as to better observe and distinguish subtle metabolic differences between groups. After peak matching, data were clustered in positive and negative ion modes respectively and outlier samples were removed to obtain unsupervised PCA score plots and supervised PLS-DA plots. The method of 200 permutation tests was used to verify whether the PLS-DA model constructed by NC group, ALI group and each administration group had over-fitting under positive and negative ion modes. The results were shown in Figure 2.

As shown in Figures 2A-D, in PCA and PLS-DA score plots in the positive and negative ion modes were show that, the samples of NC and ALI were concentrated in different areas, and were clearly distinguished. The samples of RA-M were partially overlapped with those of RA-H and RA-L, both RA-H and RA-M were completely separated from ALI, and RA-H and NC were partially overlapped. These results indicate that the metabolic pattern of CCl_4_-induced acute liver injury model rats deviates from the normal state. Due to individual differences within the group, the metabolic profile of the middle-dose overlapped with the low-dose part and crossed with the high-dose part at the same time. However, the metabolic profile of low-dose group was completely separated from that of high-dose group due to the different dosage. The results showed that rhubarb anthraquinone could reverse the characteristics of the metabolic profiles in rats with acute liver injury, and showed a dose-dependent phenomenon. With the increase of dosage, the metabolic profile fluctuated more strongly and approached the normal group.

The validation method was used to evaluate the validity of the explicable variables (R_2_X, R_2_Y) and the model’s predictability (Q_2_) of the supervised PLS-DA model. Under positive and negative ion modes, the R_2_X (cum) values of PLS-DA were 0.725 and 0.787, R_2_Y (cum) were 0.696 and 0.732, and Q_2_ (cum) were 0.742 and 0.751, respectively. The results showed that the PLS-DA model was stable and reliable. It could be used as a preliminary analysis of the overall changes and differences in plasma samples.

As shown in Figures 2E, F, the PLS-DA model had a good discrimination degree and prediction rate, and there was no “over-fitting”. The model is reliable, which indicated that the endogenous metabolites of rhubarb anthraquinone were significantly different from those of ALI.

### OPLS-DA analysis and potential biomarkers exploring

**FIGURE 3:**
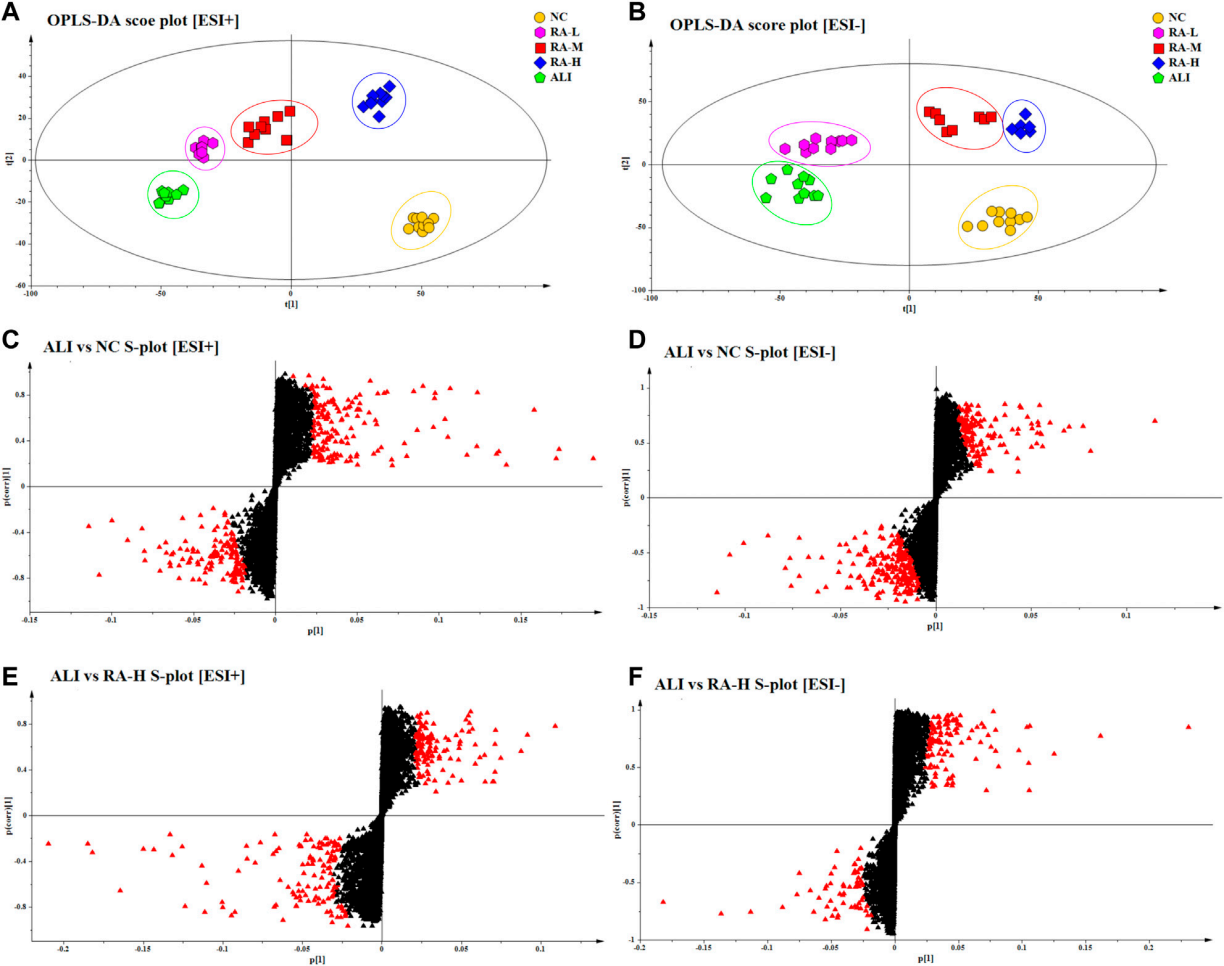
OPLS-DA score plots of different groups in ESI+ and ESI- mode respectively. The score plots of OPLS-DA of NC, ALI, RA-L, RA-M and RA-H from OPLS-DA analysis in ESI+ **(A)** and ESI- **(B)**; Metabolites with VIP >1 of ALI vs. NC were marked with a red triangle in ESI+ **(C)** and ESI- **(D)**; Metabolites with VIP >1 of ALI vs. RA-H were marked with a red triangle in ESI+ **(E)** and ESI- **(F)**.

Through PCA and PLS-DA analysis, the overall changes and differences of plasma samples were preliminarily analyzed. However, there were still some samples that cross between groups. In order to find out the different metabolites between the groups, supervised OPLS-DA was used to analyze the samples. Combined with the VIP value obtained by OPLS-DA and the S-plot constructed by OPLS-DA analysis, *p* < 0.05, VIP>1 and fold change (FC) > 2 were used as the criteria to screen metabolites of the differential map. The results were shown in Figure 3.

### The identification and explanation of potential biomarkers

**FIGURE 4:**
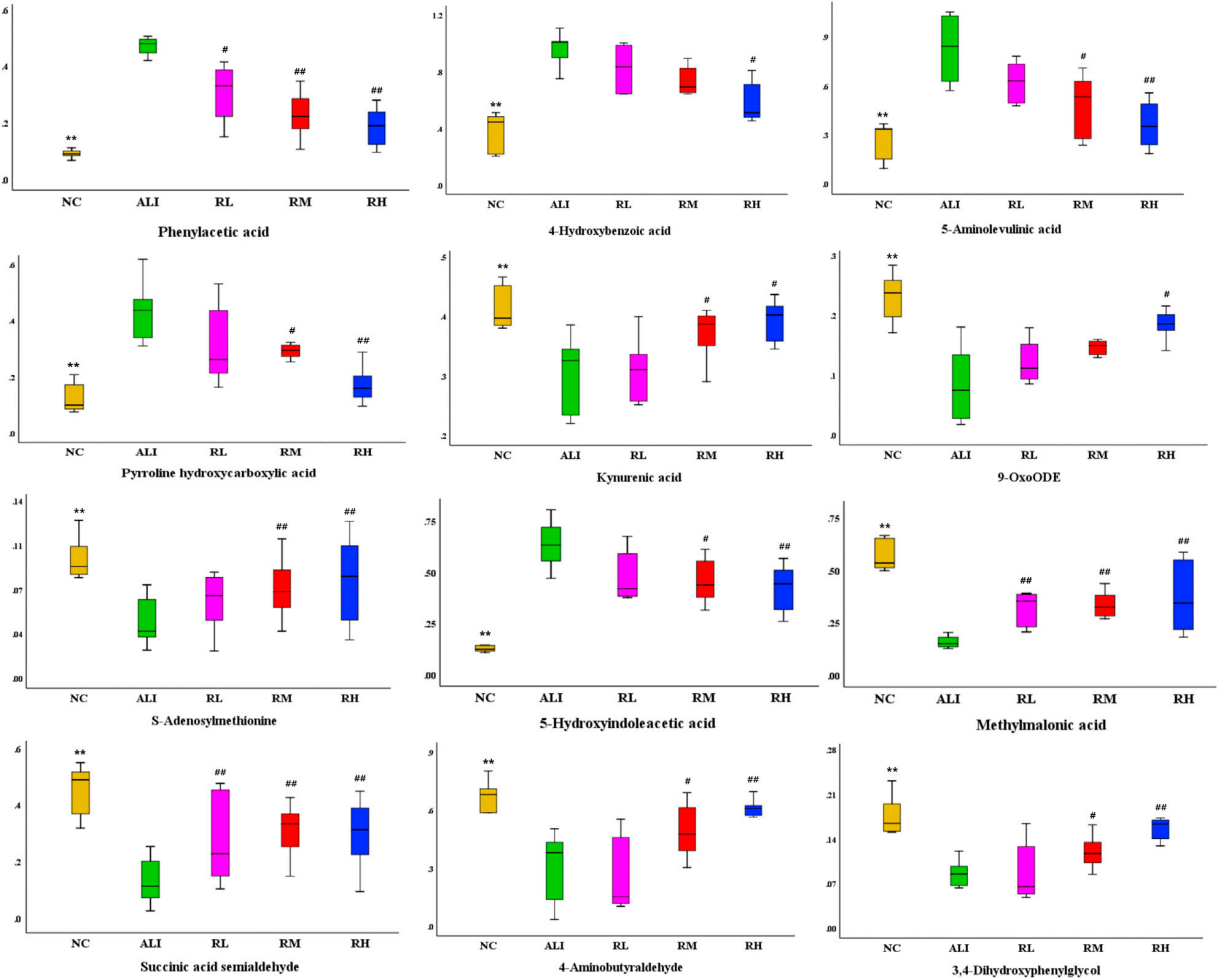
Variations in the trends of the difference metabolites. There were shown the variations in the trends of Phenylacetic acid, 4-Hydroxybenzoic acid, 5-Aminolevulinic acid, Pyrroline hydroxycarboxylic acid, Kynurenic acid, 9-OxoODE, S-Adenosylmethionine, 5-Hydroxyindoleacetic acid, Methylmalonic acid, Succinic acid semialdehyde, 4-Aminobutyraldehyde, 3, 4- Dihydroxyphenylglycol respectively. Compared with the NC group, *p < 0.05, **p < 0.01; Compared with the ALI group, ^#^ *p* < 0.05, ^##^ *p <* 0.01.

Through OPLS-DA multivariate statistical analysis and non-parametric analysis of variance, the accurate mass number and MS/MS secondary fragment information of the differential metabolites were screened, combined with HMBD, LMGP, KEGG, ChemSpider and other databases to identify the structure of metabolites, 12 kinds of characteristic metabolites were obtained. The results were shown in Table 4. The variation trend of each metabolite in different groups were shown in Figure 4.

**TABLE 4:** List of the identification of potential bio-markers among groups.

No.	Rt/min	Actual_M	Type	ppm	Proposed compound	Formula	KEGG ID
1	1.25	137.1312	[M + H]+	6	Phenylacetic acid	C_8_H_8_O_2_	C07086
2	1.78	137.0235	[M-H]-	3	4-Hydroxybenzoic acid	C_7_H_6_O_3_	C00156
3	5.50	132.0648	[M + H]+	4	5-Aminolevulinic acid	C_5_H_9_NO_3_	C00430
4	6.90	130.0487	[M + H]+	8	Pyrroline hydroxycarboxylic acid	C_5_H_7_NO_3_	C04281
5	7.16	190.0486	[M + H]+	6	Kynurenic acid	C_10_H_7_NO_3_	C01717
6	10.21	293.2128	[M-H]-	2	9-OxoODE	C_18_H_30_O_3_	C14766
7	13.83	397.2251	[M-H]-	3	S-Adenosylmethionine	C_15_H_22_N_6_O_5_S	C00019
8	14.58	191.1068	[M-H]-	1	5-Hydroxyindoleacetic acid	C_10_H_9_NO_3_	C05635
9	16.35	101.0707	[M + H]+	5	Methylmalonic acid	C_4_H_6_O_4_	C02170
10	16.43	102.0333	[M + H]+	7	Succinic acid semialdehyde	C_4_H_6_O_3_	C00232
11	17.33	88.0755	[M + H]+	1	4-Aminobutyraldehyde	C_4_H_9_NO	C02903
12	17.45	171.0335	[M + H]+	5	3, 4- Dihydroxyphenylglycol	C_8_H_10_O_4_	C05580

### Biological pathway analysis

**FIGURE 5:**
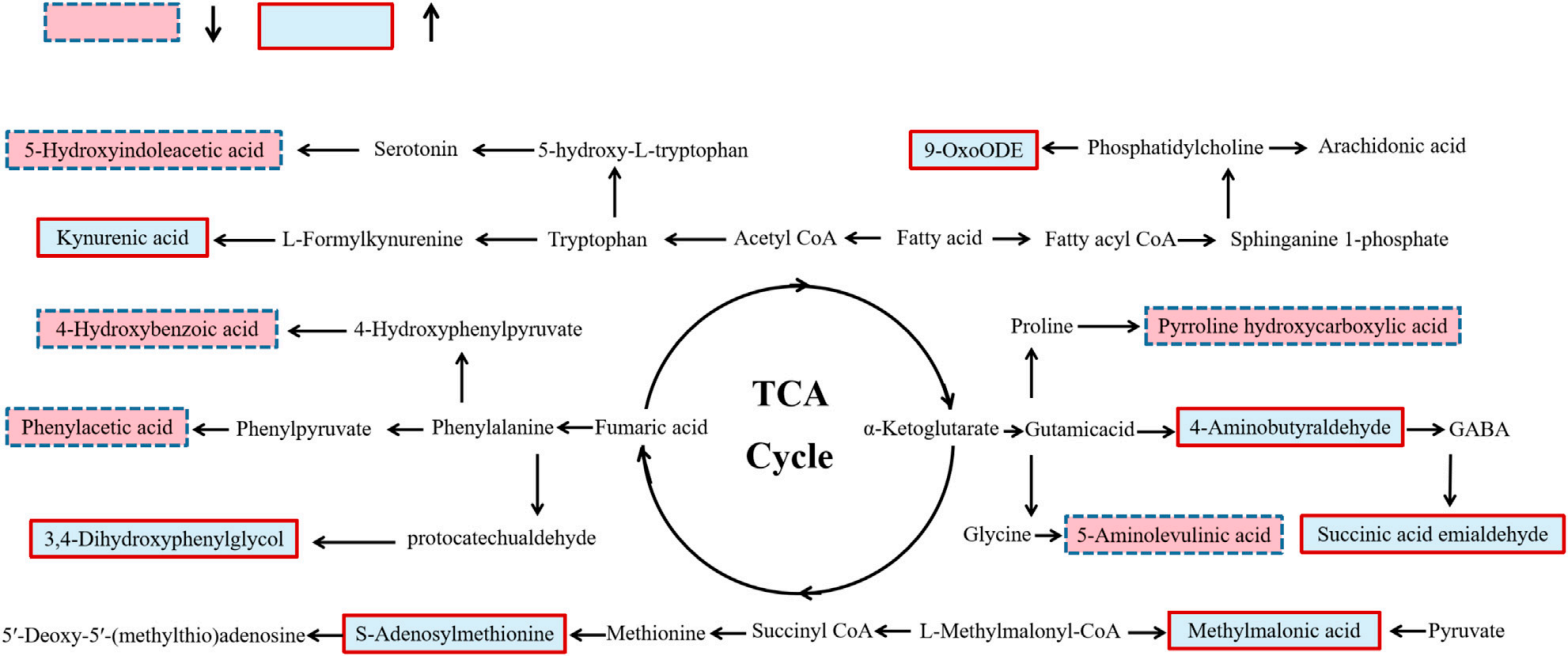
Schematic diagram of metabolic pathway related to rhubarb anthraquinone treated rat acute liver injury. The boxes bordered in red represent metabolites that are significantly higher in NC, RA-L, RA-M and RA-H than in ALI, respectively. The light blue boxes indicate metabolites significantly higher in ALI than in NC, RA-L, RA-M and RA-H respectively.

Based on metabolic biomarkers, the metabolic pathway of rhubarb anthraquinone in protecting liver was analyzed, and the KEGG metabolic pathways associated with characteristic metabolites were obtained, including the biosynthesis and metabolism of phenylalanine, tyrosine and tryptophan, the metabolism of amino sugars, nucleotide sugars and pyrimidines, and the biosynthesis of steroid hormones. According to the correlation between the metabolic pathways, the anti-acute liver injury metabolic pathway map of rhubarb anthraquinone was drawn. The results are shown in Table 5 and Figure 5.

**TABLE 5:** Metabolism-based analysis of metabolic pathways involved in the metabolism of characteristic metabolites.

No.	Pathway name	Match Status	Raw p	-log(p)	Impact	Details
1	Arginine and proline metabolism	2/38	0.03477	1.4588	0.05278	KEGG
2	Phenylalanine metabolism	1/12	0.091683	1.0377	0.0	KEGG
3	Butanoate metabolism	1/15	0.11337	0.9455	0.03175	KEGG
4	Alanine, aspartate and glutamate metabolism	1/28	0.20195	0.6947	0.04808	KEGG
5	Porphyrin and chlorophyll metabolism	1/30	0.21484	0.6678	0.02799	KEGG
6	Cysteine and methionine metabolism	1/33	0.23381	0.6311	0.05271	KEGG
7	Glycine, serine and threonine metabolism	1/34	0.24004	0.6197	0.03523	KEGG
8	Valine, leucine and isoleucine degradation	1/40	0.27645	0.5583	0.02264	KEGG
9	Tryptophan metabolism	1/41	0.28236	0.5492	0.01391	KEGG

Total cmpd was the total number of compounds in the pathway; Hits was the actually matched number from the user uploaded data; the Raw p was the original *p*-value calculated from the enrichment analysis; the Holm p was the *p*-value adjusted by Holm-Bonferroni method; the FDR p, was the *p*-value adjusted using False Discovery Rate; the Impact was the pathway impact value calculated from pathway topology analysis.

## Discussion

As a common hepatotoxic compound, CCl_4_ can induce acute liver injury models through oxidative stress and lipid peroxidation (Ingawale et al., 2014; Mohi-Ud-Din et al., 2019). In the body, CCl_4_ can induce metabolic disorders in liver tissue, mainly manifested as mitochondrial dysfunction and lipid peroxide reaction. The dysfunctional mitochondria have high oxidative capacity and produce a large amount of reactive oxygen species, which make the content ratio between reactive oxygen species and protective oxidants unbalanced, resulting in oxidative stress response; the resulting oxidative stress and inflammatory mediator factors further inducing changes in the activity of drug metabolizing enzymes in the liver, the damaged liver tissue presents a series of complex changes in pathological and biochemical characteristics, eventually leading to severe damage to liver function (Mohi-Ud-Din et al., 2019; Wang et al., 2021; Wei et al., 2021; Sheng et al., 2022).

Literature research shown that, ALT and AST in serum as the main indicators reflecting liver function, can directly reflect the damage and degree of liver cells (Xie et al., 2015; Han et al., 2019); ALP and γ-GT were specific and important markers of cholestasis because they cannot be excreted into the blood by the biliary system after liver dysfunction, which lead to the increase of serum content (Yue et al., 2014; Berköz et al., 2021). At the same time, bile excretion was blocked, bile acid, a metabolite of TC, was abnormally discharged, and negative feedback increased the content of TC in serum. With the increase of TC content in serum, the content of LDL, which is the main carrier to promote the absorption and utilization of cholesterol in peripheral tissues, obviously increased, while the content of HDL involved in the reverse transport of total cholesterol decreased due to cholestasis (Hou et al., 2019; Berköz et al., 2021). 5-hydroxyindoleacetic acid is produced through the metabolic pathway of 5-HT, which is mainly concentrated in the brain tissue, and has an influence on the toxicity of nerve cells and the transmission of neurotransmitters at the nerve terminals (Han et al., 2019; Zhang et al., 2021; Fathi et al., 2022). The results showed that, the administration of rhubarb anthraquinone could repair damaged hepatocytes, improve the mitochondrial dysfunction and cholestasis, and enhance the metabolic function of the liver, thus playing a role in protecting the liver.

Lipid peroxidation, as one of that marker of liver metabolic disorder. Literature research shown that, as a degradation product of lipid peroxidation, the increased content of MDA indicates the enhanced lipid peroxidation in liver (Zhang et al., 2019; Chen et al., 2020). On the contrary, the decreased activity of SOD and GSH indicates the decreased ability of liver to clear free radicals and hydrogen peroxidation, alleviate lipid peroxidation, and aggravate the damage of liver cells (Liu et al., 2018; Abdel-Kawy, 2021). On the contrary, the activities of SOD and GSH decreased, indicating that the ability of liver to scavenge free radicals and hydrogen peroxide and alleviate lipid peroxidation decreased, which further aggravated the damage of liver cells. (Liu et al., 2018; Abdel-Kawy, 2021). After liver injury, due to the enhanced lipid peroxidation, the integrity of mitochondrial membrane was destroyed, and chemotactic inflammatory cells enter the liver, thus releasing a series of pro-inflammatory cytokines such as TNF-a, IL-6 and IL-8, and then showed a series of immunopathological reactions. HDL, on the other hand, blocks the signal transduction that causes liver inflammation by binding with inflammatory (Teng et al., 2010; Hashmat et al., 2022). 5-Aminolevulinic acid, as the intermediate substance in heme synthesis, can promote the production of ROS in the oxidation reaction. On the contrary, both 4-aminobutyraldehyde and succinic semialdehyde were involved in the synthesis and metabolism of GABA in the liver, which can inhibit the activity of glutamate dehydrogenase and reduce the anion and hydrogen peroxide in liver mitochondria, thereby inhibiting the increase in ROS (Sugahara, 1983; Testore et al., 1995; Luca et al., 2015; Xie et al., 2015; Ravasz et al., 2017). The excessive accumulation of ROS will attack and damage liver mitochondrial DNA and mitochondrial proteins, thereby causing oxidative damage to mitochondrial and generating an oxidative stress reaction. (Liu et al., 2019; Owari et al., 2022). 4-hydroxybenzoic acid can be used as the effective scavenger of free radicals and active nitrogen substances, and was also associated with pro-oxidation and inflammation (Yoo et al., 2017; Maldonado et al., 2018). Kynuric acid as the metabolism of methylkynurenine, which has anti-inflammatory and antioxidant activities, and such activities should also be considered as the mechanism related to its hepatoprotective effect (Liu et al., 2018; Wang et al., 2019; Maitre et al., 2020). After administration of rhubarb anthraquinone, it regulates antioxidant activity, reduces the release and expression of inflammatory cytokines, alleviates oxidative stress-related damage, protects the liver, and improves liver function.

As a unique amino acid of collagen fiber, HYP content in liver tissue increases, which indicates that after liver damage, liver cells are repaired through regeneration, stimulating fibrocyte proliferation and further presenting liver fibrosis (Carter et al., 1982; Park et al., 2000; Moslemi et al., 2021). 3, 4-dihydroxyphenylethylene glycol was used as the biomarker of MAO inhibition, while MAO activity is parallel to the process of nodule formation on the surface of liver, and increased with the gradual aggravation of hepatic fibrosis (Palatý, 1985; Poirier et al., 1985); phenylacetic acid, the metabolite of phenolic acid in phenylalanine pathway, has been identified as representative substance of uremic toxin together with p-cresol sulfate and indophenol acid, which can also induce fat accumulation and lead to fat denaturation, and was considered as potential inducer of steatosis (Morita et al., 2011; Gironès et al., 2014; Saldanha et al., 2016). phenylacetic acid synthesized phenylacetyl glutamine with glutamine in liver and kidney, and then excreted from the body with urine, further reducing the toxic level of ammonia (Mokhtarani et al., 2012; Mokhtarani et al., 2013; Wang et al., 2021). After the administration of rhubarb anthraquinone, it can inhibit the irritant proliferation of liver tissue cells, inhibit the accumulation of triglycerides in the plasma of liver cells and cause liver cell necrosis, thus delaying the process of liver fibrosis.

## Conclusion

The experiment was based on the method of biochemical analysis and histopathological observation of rhubarb anthraquinone administered with different doses of acute liver injury model rats, and the results show that there is a certain correlation between the protective effect of rhubarb anthraquinone on acute liver injury and the administration dose. Within the given range, the larger the dose, the more obvious the degree of protection, which provides a reference for the clinical dosage of rhubarb. In addition, based on UPLC-MS and metabonomics, the biomarkers of rhubarb anthraquinone protecting acute liver injury in rats were screened and studied. The changes of Biological components in the body after rhubarb anthraquinone administration were analyzed by metabonomics method, which provided a research basis for the metabolic regulation mechanism of rhubarb anthraquinone in protecting acute liver injury.

## Data Availability

The original contributions presented in the study are included in the article/Supplementary Materials, further inquiries can be directed to the corresponding authors.

## References

[B1] Abdel-Kawy H. S. (2021). Effect of carvedilol versus propranolol on acute and chronic liver toxicity in rats. Drug Chem. Toxicol. 44, 101–111. 10.1080/01480545.2019.1576718 30810389

[B2] Arosio B. Gagliano N. Fusaro L. M. Parmeggiani L. Tagliabue J. Galetti P. (2000). Aloe-Emodin quinone pretreatment reduces acute liver injury induced by carbon tetrachloride. Pharmacol. Toxicol. 87, 229–233. 10.1034/j.1600-0773.2000.d01-79.x 11129503

[B3] Berköz M. Ünal S. Karayakar F. Yunusoğlu O. Özkan-Yılmaz F. Özlüer-Hunt A. (2021). Prophylactic effect of myricetin and apigenin against lipopolysaccharide-induced acute liver injury. Mol. Biol. Rep. 48, 6363–6373. 10.1007/s11033-021-06637-x 34401985

[B4] Carter E. A. McCarron M. J. Alpert E. Isselbacher K. J. (1982). Lysyl oxidase and collagenase in experimental acute and chronic liver injury. Gastroenterology 82, 526–534. 10.1016/s0016-5085(82)80402-2 6119272

[B5] Chen Y. Que R. Lin L. Shen Y. Liu J. Li Y. (2020). Inhibition of oxidative stress and NLRP3 inflammasome by Saikosaponin-d alleviates acute liver injury in carbon tetrachloride-induced hepatitis in mice. Int. J. Immunopathol. Pharmacol. 34, 2058738420950593. 10.1177/2058738420950593 32816567 PMC7444099

[B6] Chen Y. Y. Chiang S. Y. Lin J,G. Ma Y. S. Liao C. L. Weng S. W. (2010). Emodin, aloe-emodin and rhein inhibit migration and invasion in human tongue cancer SCC-4 cells through the inhibition of gene expression of matrix metalloproteinase-9. Int. J. Oncol. 36, 1113–1120. 10.3892/ijo_00000593 20372784

[B7] Crismale J. F. Friedman S. L. (2020). Acute liver injury and decompensated cirrhosis, 104. Chengdu, China: The Medical clinics of North America, 647–662. 10.1016/j.mcna.2020.02.010 32505258

[B8] Fathi M. Vakili K. Yaghoobpoor S. Tavasol A. Jazi K. Hajibeygi R. (2022). Dynamic changes in metabolites of the kynurenine pathway in alzheimer's disease, Parkinson's disease, and huntington's disease: A systematic review and meta-analysis. Front. Immunol. 13, 997240. 10.3389/fimmu.2022.997240 36263032 PMC9574226

[B9] Frank D. Savir S. Gruenbaum B. F. Melamed I. Grinshpun J. Kuts R. (2020). Inducing acute liver injury in rats via carbon tetrachloride (CCl_4_) exposure through an orogastric tube. J. Vis. Exp. JoVE. 10. 10.3791/60695 PMC7859859 32420997

[B10] Gironès N. Carbajosa S. Guerrero N. A. Poveda C. Chillón-Marinas C. Fresno M. (2014). Global metabolomic profiling of acute myocarditis caused by Trypanosoma cruzi infection. PLoS Negl. Trop. Dis. 8, e3337. 10.1371/journal.pntd.0003337 25412247 PMC4239010

[B11] Han C. Wei Y. Wang X. Ba C. Shi W. (2019). Protective effect of Salvia miltiorrhiza polysaccharides on liver injury in chickens. Poult. Sci. 98, 3496–3503. 10.3382/ps/pez153 30953070

[B12] Hashmat Z. Channa I. S. Safdar M. Ozaslan M. Saeed M. Siddique F. (2022). Adrenergic blocker terazosin potentially suppresses acetaminophen induced-acute liver injury in animal models via CYP2E1 gene. Toxicol. Res. 38, 323–330. 10.1007/s43188-021-00116-y 35874506 PMC9247125

[B13] Hou R. Liu X. Yan J. Xiang K. Wu X. Lin W. (2019). Characterization of natural melanin from Auricularia auricula and its hepatoprotective effect on acute alcohol liver injury in mice. Food and Funct. 10, 1017–1027. 10.1039/c8fo01624k 30706914

[B14] Ingawale D. K. Mandlik S. K. Naik S. R. (2014). Models of hepatotoxicity and the underlying cellular, biochemical and immunological mechanism(s): A critical discussion. Environ. Toxicol. Pharmacol. 37, 118–133. 10.1016/j.etap.2013.08.015 24322620

[B15] Li H. D. Meng X. M. Huang C. Zhang L. Lv X. W. Li J. (2019). Application of herbal traditional Chinese medicine in the treatment of acute kidney injury. Front. Pharmacol. 10, 376. 10.3389/fphar.2019.00376 31057404 PMC6482429

[B16] Li Z. Y. He P. Sun H. F. Qin X. M. Du G. H. (2014). (1)H NMR based metabolomic study of the antifatigue effect of Astragali Radix. Mol. Biosyst. 10, 3022–3030. 10.1039/c4mb00370e 25201073

[B17] Liu T. Ma X. Ouyang T. Chen H. Xiao Y. Huang Y. (2019). Efficacy of 5-aminolevulinic acid-based photodynamic therapy against keloid compromised by downregulation of SIRT1-SIRT3- SOD2-mROS dependent autophagy pathway. Redox Biol. 20, 195–203. 10.1016/j.redox.2018.10.011 30368039 PMC6205077

[B18] Liu W. Wang Z. Hou J. G. Zhou Y. D. He Y. F. Jiang S. (2018). The liver protection effects of maltol, a flavoring agent, on carbon tetrachloride-induced acute liver injury in mice via inhibiting apoptosis and inflammatory response. Molecules 23, 2120. 10.3390/molecules23092120 30142916 PMC6225187

[B19] Liu X. Yu T. Hu Y. Zhang L. Zheng J. Wei X. (2021). The molecular mechanism of acute liver injury and inflammatory response induced by Concanavalin A. Mol. Biomed. 2, 24. 10.1186/s43556-021-00049-w 35006454 PMC8607380

[B20] Liu Y. Wen P. H. Zhang X. X. Dai Y. He Q. (2018). Breviscapine ameliorates CCl_4_-induced liver injury in mice through inhibiting inflammatory apoptotic response and ROS generation. IInternational J. Mol. Med. 42, 755–768. 10.3892/ijmm.2018.3651 PMC6034936 29717768

[B21] Luca G. Vienne J. Vaucher A. Jimenez S. Tafti M. (2015). Central and peripheral metabolic changes induced by gamma-hydroxybutyrate. Sleep 38, 305–313. 10.5665/sleep.4420 25515097 PMC4288612

[B22] Lv X. M. Ma L. J. (2016). Advances in Chinese medicine treatment of acute hepatic injury. Chin. J. New Drugs 25, 170–174.

[B23] Maitre M. Klein C. Patte-Mensah C. Mensah-Nyagan A. G. (2020). Tryptophan metabolites modify brain aβ peptide degradation: A role in alzheimer's disease? Prog. Neurobiol. 190, 101800. 10.1016/j.pneurobio.2020.101800 32360535

[B24] Maldonado P. D. Chánez-Cárdenas M. E. Fernández-López A. (2018). Mechanisms of cell damage in neurological diseases and putative neuroprotective strategies. Oxidative Med. Cell. Longev. 2018, 9784319. 10.1155/2018/9784319 PMC6036792 30046382

[B25] Mohi-Ud-Din R. Mir R. H. Sawhney G. Dar M. A. Bhat Z. A. (2019). Possible pathways of hepatotoxicity caused by chemical agents. Curr. drug Metab. 20, 867–879. 10.2174/1389200220666191105121653 31702487

[B26] Mokhtarani M. Diaz G. A. Rhead W. Berry S. A. Lichter-Konecki U. Feigenbaum A. (2013). Elevated phenylacetic acid levels do not correlate with adverse events in patients with urea cycle disorders or hepatic encephalopathy and can be predicted based on the plasma PAA to PAGN ratio. Mol. Genet. metabolism 110, 446–453. 10.1016/j.ymgme.2013.09.017 PMC4108288 24144944

[B27] Mokhtarani M. Diaz G. A. Rhead W. Lichter-Konecki U. Bartley J. Feigenbaum A. (2012). Urinary phenylacetylglutamine as dosing biomarker for patients with urea cycle disorders. Mol. Genet. metabolism 107, 308–314. 10.1016/j.ymgme.2012.08.006 PMC3608516 22958974

[B28] Morita M. Yano S. Yamaguchi T. Yamauchi M. Sugimoto T. (2011). Phenylacetic acid stimulates reactive oxygen species generation and tumor necrosis factor-α secretion in vascular endothelial cells. Ther. Apher. dialysis official peer-reviewed J. Int. Soc. Apher. Jpn. Soc. Apher. Jpn. Soc. Dialysis Ther. 15, 147–150. 10.1111/j.1744-9987.2010.00887.x 21426506

[B29] Moslemi Z. Bahrami M. Hosseini E. Mansourian M. Daneshyar Z. Eftekhari M. (2021). Portulaca oleracea methanolic extract attenuate bile duct ligation-induced acute liver injury through hepatoprotective and anti-inflammatory effects. Heliyon 7, e07604. 10.1016/j.heliyon.2021.e07604 34355097 PMC8322275

[B30] Neyrinck A. M. Etxeberria U. Taminiau B. Daube G. Van Hul M. Everard A. (2017). Rhubarb extract prevents hepatic inflammation induced by acute alcohol intake, an effect related to the modulation of the gut microbiota. Mol. Nutr. food Res. 61, 1500899. 10.1002/mnfr.201500899 26990039

[B31] Owari T. Tanaka N. Nakai Y. Miyake M. Anai S. Kishi S. (2022). 5-Aminolevulinic acid overcomes hypoxia-induced radiation resistance by enhancing mitochondrial reactive oxygen species production in prostate cancer cells. Br. J. cancer 127, 350–363. 10.1038/s41416-022-01789-4 35365766 PMC9296661

[B32] Palatý V. (1985). Inhibition of monoamine oxidase by amiloride. Can. J. physiology Pharmacol. 63, 1586–1589. 10.1139/y85-261 3830356

[B33] Park E. J. Jeon C. H. Ko G. Kim J. Sohn D. H. (2000). Protective effect of curcumin in rat liver injury induced by carbon tetrachloride. J. Pharm. Pharmacol. 52, 437–440. 10.1211/0022357001774048 10813555

[B34] Poirier M. F. Lôo H. Dennis T. Le Fur G. Scatton B. (1985). Platelet monoamine oxidase activity and plasma 3,4-dihydroxyphenylethylene glycol levels during the menstrual cycle. Neuropsychobiology 14, 165–169. 10.1159/000118222 3835494

[B35] Professional Committee of Liver Disease of China Society of Integrated Traditional Chinese and Western Medicine (2019). Diagnosis and treatment Guidelines of integrated Chinese and western medicine for treating hepatic fibrosis. J. Clin. Hepatology 27, 494–504.

[B36] Ravasz D. Kacso G. Fodor V. Horvath K. Adam-Vizi V. Chinopoulos C. (2017). Catabolism of GABA, succinic semialdehyde or gamma-hydroxybutyrate through the GABA shunt impair mitochondrial substrate-level phosphorylation. Neurochem. Int. 109, 41–53. 10.1016/j.neuint.2017.03.008 28300620

[B37] Riordan S. M. Williams R. (2003). Mechanisms of hepatocyte injury, multiorgan failure, and prognostic criteria in acute liver failure. Seminars liver Dis. 23, 203–215. 10.1055/s-2003-42639 14523674

[B38] Saldanha J. F. Yi D. Stockler-Pinto M. B. Soula H. A. Chambert S. Fouque D. (2016). Determination of the binding properties of the uremic toxin phenylacetic acid to human serum albumin. Biochimie 125, 53–58. 10.1016/j.biochi.2016.03.002 26945842

[B39] Sheng C. Guo Y. Ma J. Hong E. K. Zhang B. Yang Y. (2022). Metabolomic profiling reveals protective effects and mechanisms of sea buckthorn sterol against carbon tetrachloride-induced acute liver injury in rats. Mol. (Basel, Switz. 27, 2224. 10.3390/molecules27072224 PMC9000363 35408620

[B40] Su Y. W. Tan E. Zhang J. You J. L. Liu Y. Liu C. (2014). Study on three different species Tibetan medicine sea buckthorn by 1H-NMR-based metabonomics. Zhongguo Zhong Yao Za Zhi 39, 4234–4239. 25775800

[B41] Sugahara M. (1983). 4-Aminobutyraldehyde as a precursor convertible to gamma-aminobutyric acid *in vivo* . J. Biochem. 93, 1337–1342. 10.1093/oxfordjournals.jbchem.a134268 6885726

[B42] Teng Y. Sun C. H. Li G. Sun G. Nomachi Y. Yokota J. (2010). Protective effects of Flos lonicera extract on acute liver injury by dimethylnitrosamine-induced in rats. J. Nat. Med. 64, 288–294. 10.1007/s11418-010-0405-x 20306146

[B43] Testore G. Colombatto S. Silvagno F. Bedino S. (1995). Purification and kinetic characterization of gamma-aminobutyraldehyde dehydrogenase from rat liver. Int. J. Biochem. Cell. Biol. 27, 1201–1210. 10.1016/1357-2725(95)00075-z 7584606

[B44] Thawley V. (2017). Acute liver injury and failure. Small Anim. Pract. 47, 617–630. 10.1016/j.cvsm.2016.11.010 28065578

[B45] Wang L. S. Zhang M. D. Tao X. Zhou Y. F. Liu X. M. Pan R. L. (2019). LC-MS/MS-based quantification of tryptophan metabolites and neurotransmitters in the serum and brain of mice. J. Chromatogr. B, Anal. Technol. Biomed. life Sci. 1112, 24–32. 10.1016/j.jchromb.2019.02.021 30836315

[B46] Wang M. F. Zhao S. S. Thapa D. M. Song Y. L. Xiang Z. (2021). Metabolomics of Fuzi-Gancao in CCl_4_induced acute liver injury and its regulatory effect on bile acid profile in rats. World J. gastroenterology 27, 6888–6907. 10.3748/wjg.v27.i40.6888 PMC8567467 34790013

[B47] Wang X. Tseng J. Mak C. Poola N. Vilchez R. A. (2021). Exposures of phenylacetic acid and phenylacetylglutamine across different subpopulations and correlation with adverse events. Clin. Pharmacokinet. 60, 1557–1567. 10.1007/s40262-021-01047-5 34125423 PMC8613126

[B48] Wei B. Yang Z. D. Shi D. F. Yao X. J. Wang M. G. (2016). Inhibition of monoamine oxidase by stilbenes from rheum palmatum. Iran. J. Pharm. Res. 15, 885–892. 28243286 PMC5316268

[B49] Wei X. Luo C. He Y. Huang H. Ran F. Liao W. (2021). Hepatoprotective effects of different extracts from triphala against CCl4 -induced acute liver injury in mice. Front. Pharmacol. 12, 664607. 10.3389/fphar.2021.664607 34290606 PMC8287969

[B50] Wu Z. Han M. Chen T. Yan W. Ning Q. (2010). Acute liver failure: Mechanisms of immune-mediated liver injury. Liver Int. official J. Int. Assoc. Study Liver 30, 782–794. 10.1111/j.1478-3231.2010.02262.x 20492514

[B51] Xie J. Liu J. Chen T. M. Lan Q. Zhang Q. Y. Liu B. (2015). Dihydromyricetin alleviates carbon tetrachloride-induced acute liver injury via JNK-dependent mechanism in mice. World J. gastroenterology 21, 5473–5481. 10.3748/wjg.v21.i18.5473 PMC4427668 25987769

[B52] Xie Z. X. Xia S. F. Qiao Y. Shi Y. H. Le G. W. (2015). Effect of GABA on oxidative stress in the skeletal muscles and plasma free amino acids in mice fed high-fat diet. J. animal physiology animal Nutr. 99, 492–500. 10.1111/jpn.12254 25266692

[B53] Xue J. H. Chen F. Wang J. Wu S. S. Zheng M. Zhu H. H. (2015). Emodin protects against concanavalin A-induced hepatitis in mice through inhibiting activation of the p38 MAPK-NF-κB signaling pathway. Cell. Physiol. Biochem. 35, 1557–1570. 25792514 10.1159/000373971

[B54] Yoo S. R. Jeong S. J. Lee N. R. Shin H. K. Seo C. S. (2017). Simultaneous determination and anti-inflammatory effects of four phenolic compounds in Dendrobii Herba. Nat. Prod. Res. 31, 2923–2926. 28281361 10.1080/14786419.2017.1300798

[B55] Yue S. Hu B. Wang Z. Yue Z. Wang F. Zhao Y. (2014). Salvia miltiorrhiza compounds protect the liver from acute injury by regulation of p38 and NFκB signaling in Kupffer cells. Pharm. Biol. 52, 1278–1285. 10.3109/13880209.2014.889720 25026357

[B56] Zhang C. E. Niu M. Li R. Y. Feng W. W. Ma X. Dong Q. (2016). Untargeted metabolomics reveals dose-response characteristics for effect of rhubarb in a rat model of cholestasis. Front. Pharmacol. 7, 85. 10.3389/fphar.2016.00085 27065293 PMC4814850

[B57] Zhang C. Y. Zhu J. Y. Ye Y. Zhang M. Zhang L. J. Wang S. J. (2017). Erhuang Formula ameliorates renal damage in adenine-induced chronic renal failure rats via inhibiting inflammatory and fibrotic responses. Biomed. Pharmacother. 95, 520–528. 10.1016/j.biopha.2017.08.115 28866419

[B58] Zhang W. Y. Hu X. F. Wan N. Zhang J. F. Yang P. Wen Q. (2019). Protective effect of the glucagon-like peptide-1 analogue liraglutide on carbon tetrachloride-induced acute liver injury in mice. Biochem. Biophys. Res. Commun. 5, 386–392. 10.1016/j.bbrc.2019.04.160 31047638

[B59] Zhang Y. X. Li C. Liang X. R. Jin J. Q. Zhang Y. Xu F. (2021). Role of 5-HT degradation in acute liver injury induced by carbon tetrachloride. Eur. J. Pharmacol. 908, 174355. 10.1016/j.ejphar.2021.174355 34280394

[B60] Zhang Z. H. Vaziri N. D. Wei F. Cheng X. L. Bai X. Zhao Y. Y. (2016). An integrated lipidomics and metabolomics reveal nephroprotective effect and biochemical mechanism of Rheum officinale in chronic renal failure. Sci. Rep. 6, 22151. 10.1038/srep22151 26903149 PMC4763304

