# Supplementary figures and images for: Study on the mechanism of acute liver injury protection in Rhubarb anthraquinone by metabolomics based on UPLC-Q-TOF-MS
Source: Front Pharmacol. 2023 Mar 6;14:1141147. doi: 10.3389/fphar.2023.1141147 (PMC10025310; doi:10.3389/fphar.2023.1141147)

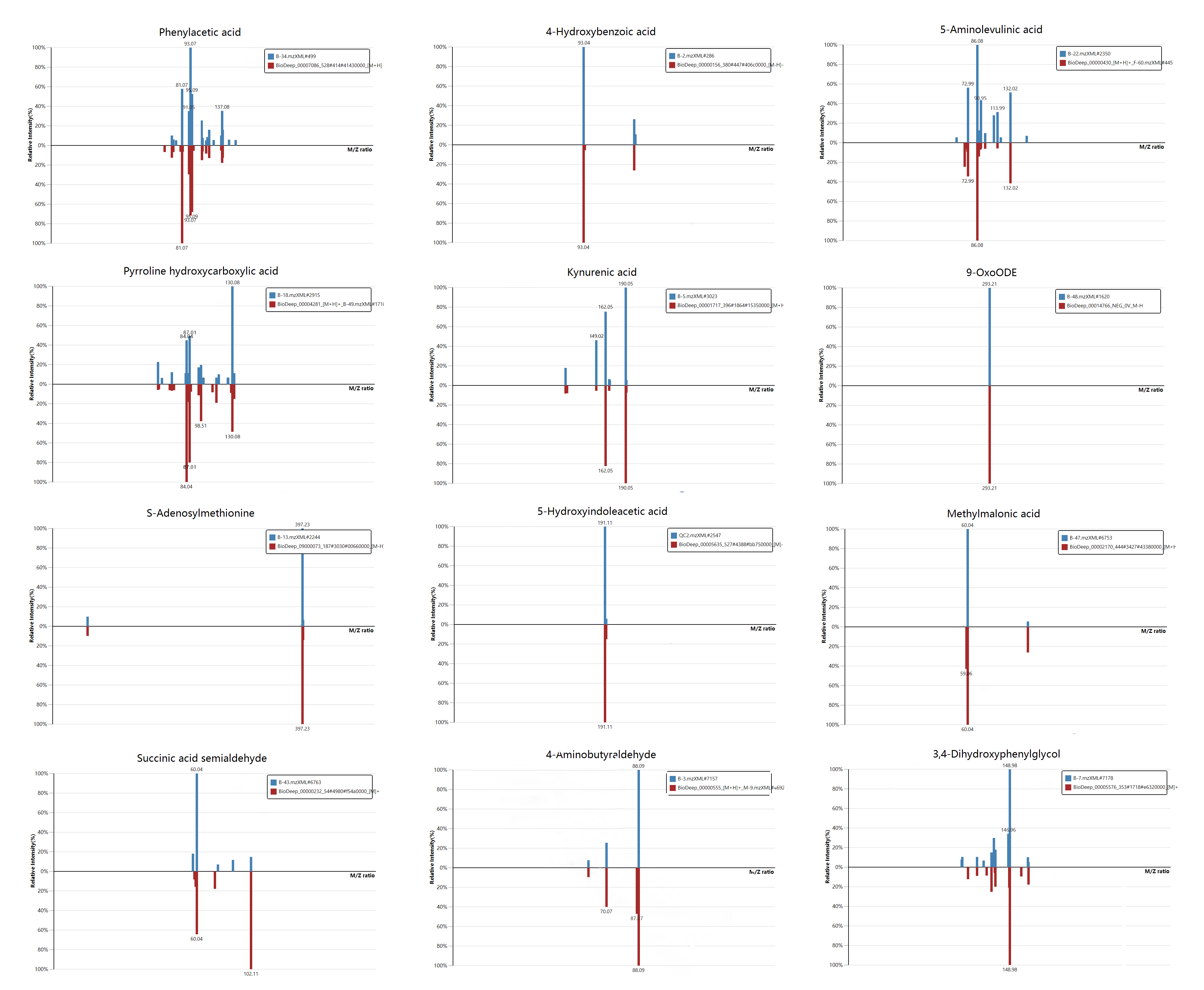

Supplement: Supplementary file 1 [file Image2.tif]

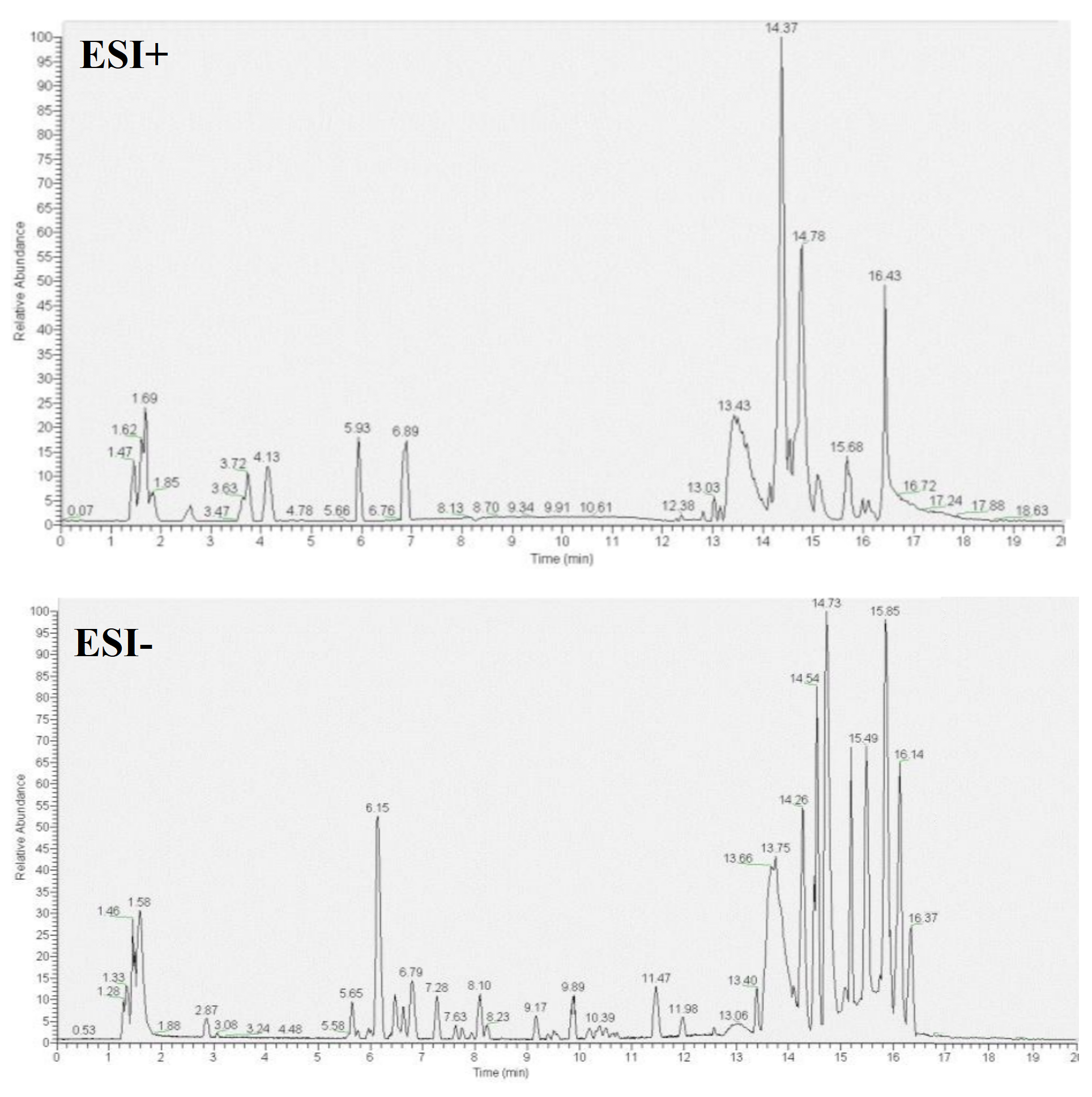

Supplement: Supplementary file 2 [file Image1.tif]
